# The Effect of N Fertilizer Placement on the Fate of Urea-^15^N and Yield of Winter Wheat in Southeast China

**DOI:** 10.1371/journal.pone.0153701

**Published:** 2016-04-15

**Authors:** Zhaoming Chen, Huoyan Wang, Xiaowei Liu, Yongzhe Liu, Shuaishuai Gao, Jianmin Zhou

**Affiliations:** 1 State Key Laboratory of Soil and Sustainable Agriculture, Institute of Soil Science, Chinese Academy of Sciences, Nanjing 210018, China; 2 University of Chinese Academy of Sciences, Beijing 100049, China; Chinese Academy of Sciences, CHINA

## Abstract

A field micro-plot experiment using nitrogen isotope (^15^N) labeling was conducted to determine the effects of placement methods (broadcast and band) and N rates (60, 150 and 240 kg ha^–1^) on the fate of urea-^15^N in the wheat–soil system in Guangde County of Anhui Province, China. N fertilizer applied in bands increased grain yield by 15% compared with broadcast application. The N fertilizer application rate had a significant effect on grain yield, straw yield and aboveground biomass, as well as on N uptake and N concentration of wheat. The recovery of urea-^15^N was a little higher for broadcast (34.0–39.0%) than for band treatment (31.2–38.2%). Most of the soil residual N was retained in the 0–20 cm soil layer. At the N rates of 60 and 240 kg ha^–1^, the residual ^15^N was higher for band (34.4 and 108.7 kg ha^–1^, respectively) than for broadcast application (29.6 and 88.4 kg ha^–1^, respectively). Compared with broadcast treatment, banded placement of N fertilizer decreased the N loss in the wheat–soil system. Band application one time is an alternative N management practice for winter wheat in this region.

## Introduction

Among the fertilizers, nitrogen (N) is the most important nutrient for wheat (*Triticum aestivum* L.) and can improve grain yield and quality of winter wheat [1, 2]. To obtain high yields of cereal crops, high rates of N fertilizer, especially manufactured N fertilizer, are applied by farmers in China [3]. However, the increase of crop yields is not proportion with the large increase of consumption of N fertilizer [4, 5]. Excess inorganic N and inappropriate application methods have led to low N use efficiency (NUE) and negative environmental impacts [6–9]. Efficient N fertilizer management is vital to maintain wheat production and soil fertility, and minimize environmental risk [10–12].

Efficient use of N fertilizer depends very much on timing, rates, resources and placement of N fertilizer application [13]. The placement method of fertilizer N influences crop yield and N uptake by cereal crops [14, 15]. Split application of N fertilizer has been shown to improve the yield, grain protein concentration and NUE of wheat, compared to a single surface application [16]. The uptake of surface-applied fertilizer depends on the following rain or irrigation [17], and in fields with limited rain or irrigation, broadcast-applied N fertilizer remains near the soil surface and does not move into the root zone where it can be absorbed by plants [18]. Compared with placement of N fertilizer on the soil surface, N fertilizer applied in bands can reduce N loss via ammonia volatilization and improve NUE of plants [19]. Banding of urea leads to high ammonium (NH_4_^+^) concentration in the fertilizer zone that can inhibit nitrifying bacteria, and so reduce N loss due to leaching [15, 20, 21]. Additionally, placing N fertilizer into soil can reduce the immobilization of N by soil microorganisms and increases uptake by plants [22, 23]. Banding of N fertilizer can also improve the competition of wheat with weeds compared to broadcast [18].

Appropriate N application rate is an important N management practice for winter wheat [24]. The increasing rate of wheat yield is not linear with increasing rate of N application; for example, when N was applied at 120 and 180 kg ha^–1^, the corresponding wheat yields were 3600 and 3000 kg ha^–1^ [1]. The economic yield of winter wheat is a maximum at the N rate of 210 kg ha^–1^ in Jiangsu Province, China [9]. The N recovery in wheat increased with increasing N rates in a pot experiment [25]. However, other researchers reported that the NUE of wheat decreased with increasing rate of N application under field conditions [24, 26]. Future increases in crop production must be achieved through improved N efficiency rather than increased N rates [27].

Band application one time of N fertilizer would not only increase wheat grain yield, but also decrease the N loss, and still is time-saving. However, little information about effects of band application one time of N fertilizer on winter wheat in southeast China. The ^15^N tracer technique has been widely used to distinguish the plant N from fertilizer or soil N and the N fluxes in agroecosystems [26, 28]. This study aimed to determine the fate of urea-^15^N in the wheat–soil system as influenced by placement method and N rate in the Middle and Lower Yangtze River basin in southeast China, during 2014–2015.

## Materials and Methods

### Site Description

**Fig 1:**
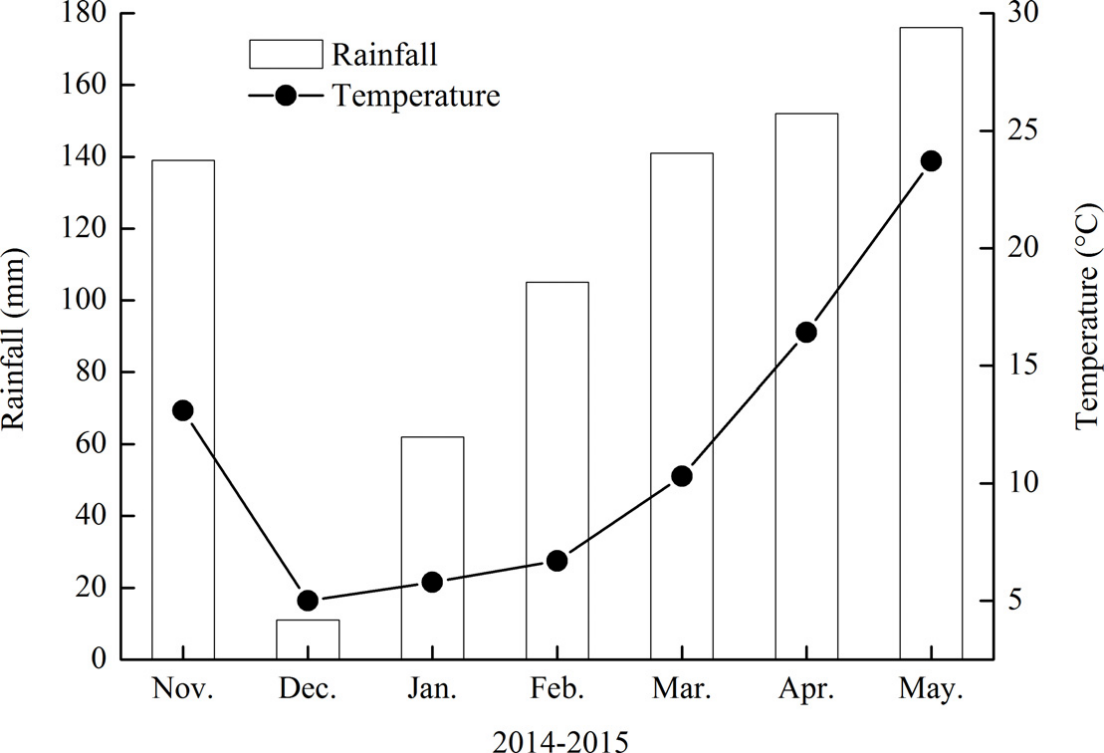
The monthly mean rainfall and mean temperature during the wheat growing season from 2014 to 2015.

The field micro-plot experiment was conducted in Guangde County of Anhui Province (31°01ʹN, 119°26′E), China, during 2014–2015. The farmer had gave us permission to conduct the study in the site prior to the start of the experiment. The region is classified as sub-tropical with a monsoon climate. The soil at Guangde is an Alfisol (FAO classification), with topsoil (0–20 cm) properties as follows: soil bulk density 1.02 g cm^–3^, total carbon (C) 20.58 g kg^–1^, total N 1.12 g kg^–1^, Olsen- phosphorous (P) 25.51 mg kg^–1^, available potassium (K) 65.00 mg kg^–1^ and pH (water) 5.52. The rainfall and temperature data during the wheat season are shown in Fig 1. Rice–winter wheat rotation is used in the site. Total rainfall during the winter wheat growing season was 786 mm (Fig 1). Our study did not involve the endangered or protected species.

### Experiment Design

The micro-plots (0.5 m × 0.5 m) were bounded by plastic frames of 0.35 m high to monitor the fates of ^15^N-labeled fertilizer. The frames were pressed 0.3 m deep into the soil and protruded 0.05 m above the soil to prevent runoff and lateral contamination. The experimental design was a randomized complete block design with three replications. Two rows of wheat were planted in each micro-plot with row spacing of 25 cm. A local wheat cultivar, Yangmai 20, was sown in early November 2014 with the seed rate of 187.5 kg ha^–1^ and was harvested in early June 2015. The experiment was a factorial design with two placement methods (broadcast and band) and three N rates of 60, 150 and 240 kg N ha^–1^ termed N 60, N 150 and N 240, respectively. For fertilizer N banding, the ^15^N-labeled urea as basal fertilizer was side-banded by shovel 5 cm from wheat and 10 cm under the soil surface at one time. For fertilizer broadcasting, the ^15^N-labeled urea was broadcast and incorporated into soil at sowing, and was broadcast on soil surface at tillering and at jointing in split applications (with a ratio of base-N to topdress-N of 1:0.75:0.75). Wheat was planted around the micro-plot to reduce edge effects, with the placement method and N rate the same as in the corresponding micro-plot. The ^15^N-labeled urea (^15^N abundance was 10.15%) was provided by Shanghai Research Institute of Chemical Industry. The P fertilizer (150 kg P_2_O_5_ ha^–1^ as triple superphosphate) and K fertilizer (180 kg K_2_O ha^–1^ as potassium chloride) were applied to all micro-plots at sowing. No irrigation was applied due to abundant rainfall in the winter wheat growing season (Fig 1).

### Plant and Soil Sampling and ^15^N Analyses

Plants were harvested by hand in each micro-plot after wheat maturity. Plant aboveground parts were divided into grain and straw. Roots of each micro-plot were all collected from the 0–20 cm soil layer and washed. The fresh grain, straw and root samples were dried at 75°C to constant weight for determination of dry weight. The dry samples were ground to powder and passed through a 150-μm screen before N content and ^15^N analysis.

Soils were sampled to a depth of 80 cm in 20-cm increments. The method of collecting soil sample for the banding treatment was described by Jayasundara et al. [29, 30]. Four soil cores (5 cm in diameter) were collected for the banding treatment: two from the fertilizer band and the others from the middle of two wheat rows. The same depth segments of four soil cores were mixed well as a single soil sample. The soil samples under broadcast treatment were randomly collected from four points and the same depth segments were mixed well as a single soil sample. The soil samples were air-dried and ground to pass through a 150-μm screen for ^15^N analysis. Soil bulk density was determined after harvesting the wheat using the cutting ring method [31]. Grain, straw, root and soil samples were analyzed for total N and ^15^N abundance using an elemental analyzer (Costech ECS4010, Costech Analytical Technologies Inc., Valencia, USA) coupled to an isotope ratio mass spectrometer (Delta V Advantage, Thermo Fisher Scientific Inc., USA).

### Calculation Methods

All ^15^N was expressed as the atom percent excess corrected for background abundance (i.e. 0.366%). The bulk densities of 0–20, 20–40, 40–60 and 60–80 cm soil layers were 1.02, 1.17, 1.23 and 1.21 g cm^–3^, respectively.

The amounts of plant N derived from fertilizer (Ndff) and from soil (Ndfs), and the soil residual N were calculated as follows [16].

$$\mathrm{Ndff}\;(\%)=\frac{B-A}{C-A}\;\times \;100$$ (1)

where A is the ^15^N natural abundance, B is the ^15^N atom percent excess in the plant or soil and C is the ^15^N atom percent excess in the fertilizer N.

$$\mathrm{Grain Ndff}\;(\mathrm{kg}\;ha^{-1})=N\;\mathrm{uptake by grain}\;\times \;\left(1\right)$$ (2)

$$\mathrm{Straw Ndff}\;(kg\;ha^{-1})=\mathrm{N uptake by straw}\;\times \;\left(1\right)$$ (3)

$$\mathrm{Root Ndff}\;(kg\;ha^{-1})=\mathrm{N uptake by root}\;\times \;\left(1\right)$$ (4)

$$\mathrm{Plant Ndff}\;(kg\;ha^{-1})=(2)+(3)+(4)$$ (5)

$$\begin{array}{l} Soil\;residual\;N\;(kg\;ha^{–1})=total\;soil\;N\;\times \;soil\;bulk\;density\\ \;\times \;soil\;thickness\;\times \;\left(1\right) \end{array}$$ (6)

### Statistical Analysis

Statistical analysis was conducted using SPSS 16.0 (SPSS Inc., Chicago, USA) for analysis of variance (ANOVA). Two-way ANOVA was conducted to assess the effects of N fertilizer placement and N rate on wheat yield, N uptake and fate of urea-^15^N. Significances among six treatments were compared by the least significance difference at *P* < 5% level.

## Results

### Crop Biomass

Placement method and N rate significantly affected grain yield, straw yield and aboveground biomass of winter wheat (Table 1). The grain yield of wheat was higher for band (3.83–5.42 t ha^–1^) than for broadcast treatment (3.32–4.74 t ha^–1^). The highest grain yield of wheat was 4.74 t ha^–1^ at N 240 for broadcast treatment and 5.42 t ha^–1^ at N 240 for band treatment. Both straw yield and aboveground biomass were also higher for band (mean of 6.57 and 11.4 t ha^–1^, respectively) than for broadcast treatment (mean of 5.15 and 9.34 t ha^–1^, respectively). The root biomass of winter wheat was similar across all treatments. Placement method significantly affected the harvest index (HI) of wheat. The HI was lower for band (0.41–0.43) than for broadcast treatment (0.44–0.46). This indicated that the increase of grain yield was not linear with increased aboveground biomass. The grain yield, straw yield and total aboveground biomass increased with increasing rate of N application, respectively (*P* < 0.001). The interaction of placement and N rate only significantly affected the HI (*P* < 0.05).

**Table 1:** Effects of N fertilizer placement and N rate on grain yield, straw yield, aboveground biomass, root biomass and harvest index (HI) of winter wheat.

Treatment		Dry matter yield (t ha^-1^)				Harvest index
Placement	N rate	Grain	Straw	Aboveground	Root	
Broadcast	N 60	3.32e	4.25e	7.57d	0.93	0.44bc
	N 150	4.53c	5.43cd	9.96bc	0.91	0.46a
	N 240	4.74bc	5.76c	10.5b	0.95	0.45ab
Band	N 60	3.83d	5.05d	8.89c	1.00	0.43c
	N 150	5.21ab	6.97b	12.2a	0.94	0.43c
	N 240	5.42a	7.68a	13.1a	0.98	0.41d
Treatment effects						
Placement (P)		***	***	***	NS	***
N rate (N)		***	***	***	NS	NS
P×N		NS	NS	NS	NS	*

Different letters within a column represent significant differences (*P* < 0.05).

***, * Significant at *P* < 0.001 and *P* < 0.05, respectively; NS not significant.

### N Concentration and N Uptake

The grain N concentration was higher at N 60, N 150 and N 240 under broadcast treatment (15.3, 18.3 and 22.5 g kg^–1^, respectively) than the corresponding N rates under band treatment (14.4, 15.3 and 18.3 g kg^–1^), but the grain yield was higher under band treatment, compared to broadcast treatment (Tables 1 and 2). This resulted in a similar N uptake in grain by wheat for broadcast and band treatments at the same N application rate (Table 2). The N concentration in straw and roots were not significantly affected by placement method. The wheat N concentration and N uptake increased with increasing N rate (*P* < 0.01) (Table 2). The N application method had no effect on the N uptake by wheat, but did affect the N uptake in straw. At N 150, the N uptake in straw was higher for band (22.6 kg ha^–1^) than for broadcast treatment (17.7 kg ha^–1^). The total N uptake by wheat was within the range of 66.5–136.3 kg ha^–1^ (mean 102.4 kg ha^–1^) for broadcast and 72.1–133.0 kg ha^–1^ (mean 104.1 kg ha^–1^) for band treatments.

**Table 2:** Effects of N fertilizer placement and N rate on N content and N accumulation in grain, straw and roots of winter wheat.

Treatment		N concentration (g kg^-1^)			N uptake (kg ha^-1^)			
Placement	N rate	Grain	Straw	Root	Grain	Straw	Root	Total
Broadcast	N 60	15.3c	2.90cd	3.93c	50.7c	12.2d	3.67b	66.5c
	N 150	18.3b	3.27bc	4.53bc	82.7b	17.7c	4.10b	104.5b
	N 240	22.5a	4.30a	4.83ab	106.9a	24.8ab	4.60ab	136.3a
Band	N 60	14.4c	2.57d	4.00c	55.1c	12.9d	4.03b	72.1c
	N 150	15.3c	3.27bc	4.83ab	80.0b	22.6b	4.53ab	107.2b
	N 240	18.3b	3.70b	5.40a	99.3a	28.5a	5.33a	133.0a
Treatment effects								
Placement (P)		***	NS	NS	NS	*	NS	NS
N rate (N)		***	***	***	***	***	**	***
P×N		**	NS	NS	NS	NS	NS	NS

Different letters within a column represent significant differences (*P* < 0.05).

***, **, * Significant at *P* < 0.001, *P* < 0.01 and *P* < 0.05, respectively; NS not significant.

### Distribution of Urea-^15^N and Soil N in Wheat Plants

Grain N uptake derived from ^15^N-labeled urea for broadcast and band treatments was in the range of 35.9–60.2% (mean 50.2%) and 33.2–57.0% (mean 47.0%), respectively. The total N uptake derived from fertilizer was 4–11% higher for broadcast than for band treatment. The average Ndff in grain (48.5%) and straw (46.3%) was higher than that in root (37.4%). The Ndff in wheat plant increased but Ndfs decreased with increasing N rate for both broadcast and band treatments (Table 3). The total N uptake by wheat from soil (43.1–58.2 kg ha^–1^) and that from urea-N (22.9–81.7 kg ha^–1^) increased with increasing N application rate (*P* < 0.001) (Table 4). At N 240, the Ndff in grain was higher for broadcast (64.4 kg ha^–1^) than for band treatment (56.6 kg ha^–1^). The N application method had no effect on Ndff in straw (except for N 150), roots and total plants. The highest Ndfs of straw for band treatment was 12.7 kg ha^–1^, significantly higher than the highest of 9.7 kg ha^–1^ for broadcast treatment. The total N derived from soil was within the range of 43.1–54.6 kg ha^–1^ for broadcast and 49.1–58.2 kg ha^–1^ for band treatment.

**Table 3:** Effects of N fertilizer placement and N rate on distributions of urea-^15^N in wheat plants.

Treatment		Ndff (%)				Ndfs (%)			
Placement	N rate	Grain	Straw	Root	total	Grain	Straw	Root	total
Broadcast	N 60	35.9e	35.5d	30.1c	35.2d	64.1b	66.5b	69.9a	64.8b
	N 150	53.7c	48.0c	32.4bc	51.9c	46.3d	52.0c	67.6ab	48.1c
	N 240	60.2a	60.8a	48.5a	59.9a	39.8f	39.2e	51.5c	40.1d
Band	N 60	33.2f	28.7e	23.6c	31.8e	66.8a	71.3a	76.4a	68.2a
	N 150	50.7d	49.5c	41.9ab	50.1c	49.3c	50.5c	58.1bc	49.9c
	N 240	57.0b	55.3b	48.1a	56.3b	43.0e	44.7d	51.9c	43.7e
Treatment effects									
Placement (P)		**	*	NS	**	**	*	NS	**
N rate (N)		***	***	***	***	***	***	***	***
P×N		NS	NS	NS	NS	NS	NS	NS	NS

Different letters within a column represent significant differences (*P* < 0.05).

***, **, * Significant at *P* < 0.001, *P* < 0.01 and *P* < 0.05, respectively; NS not significant.

**Table 4:** Effects of N fertilizer placement and N rate on N derived from fertilizer (Ndff) and from soil (Ndfs) of wheat plants.

Treatment		Ndff (kg ha^-1^)				Ndfs (kg ha^-1^)			
Placement	N rate	Grain	Straw	Root	Total	Grain	Straw	Root	Total
Broadcast	N 60	18.2d	4.1d	1.1d	23.4c	32.4c	8.1c	2.6a	43.1c
	N 150	44.4c	8.5c	1.3cd	54.2b	38.3ab	9.2bc	2.8a	50.2b
	N 240	64.4a	15.1a	2.2ab	81.7a	42.5a	9.7bc	2.4a	54.6ab
Band	N 60	18.3d	3.7d	0.9d	22.9c	36.8bc	9.2bc	3.1a	49.1bc
	N 150	40.6c	11.2b	1.9bc	53.7b	39.4ab	11.4ab	2.6a	53.5ab
	N 240	56.6b	15.7a	2.6a	74.9a	42.7a	12.7a	2.8a	58.2a
Treatment effects									
Placement (P)		*	NS	NS	NS	NS	**	NS	*
N rate (N)		***	***	***	***	***	***	NS	***
P×N		NS	NS	NS	NS	NS	NS	NS	NS

Different letters within a column represent significant differences (*P* < 0.05).

***, **, * Significant at *P* < 0.001, *P* < 0.01 and *P* < 0.05, respectively; NS not significant.

### Distribution of Residual Urea-^15^N in Soil

**Fig 2:**
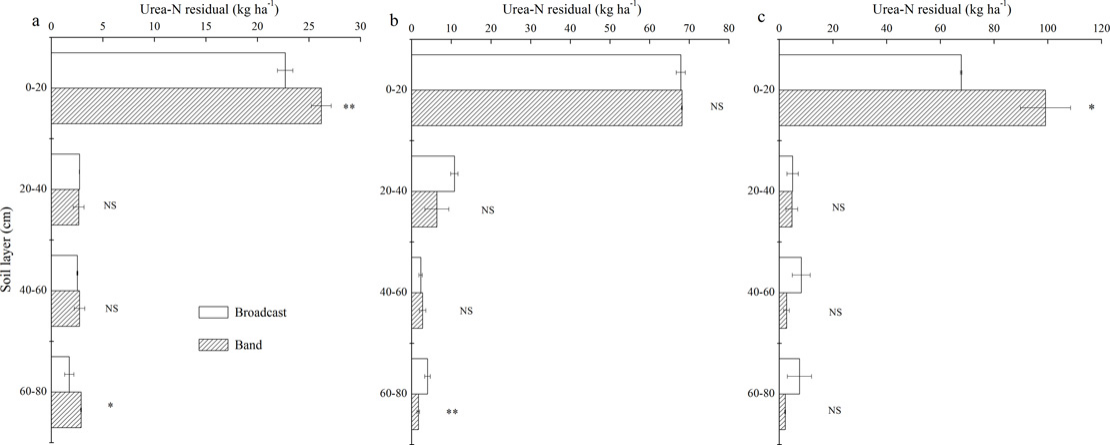
The distribution of urea-N residual in soil at (a) 60, (b) 150 and (c) 240 kg ha^-1^. ** Significant at *p*<0.01, * Significant at *p*<0.05, NS, not significant.

After winter wheat was harvested, the total residual urea-^15^N for broadcast treatment was 29.6, 84.9 and 88.4 kg ha^–1^ at N 60, N 150 and N 240, respectively, and correspondingly for band treatment was 34.4, 78.9 and 108.7 kg ha^–1^ (Fig 2). Most residual N remained in the 0–20 cm soil layer, accounting for 76.5–79.9% of total residual urea-N for broadcast and 76.0–91.2% for band application. The residual ^15^N in the 0–20 cm soil layer of band treatment for N 60 and N 240 was 26.2 and 99.1 kg ha^–1^, respectively, and was significantly (*P* < 0.05) higher than corresponding values for broadcast treatment of 22.7 and 67.7 kg ha^–1^. At N 150, there was no difference in topsoil (0–20 cm) urea-^15^N between broadcast and band treatments. In the 60–80 cm soil layer, the residual N was higher at N 60 for band than for broadcast treatment, but was lower at N 150 for band than for broadcast treatment. The 20–40- and 40–60-cm soil residual N was not significantly affected by the placement method. At higher N application rates (N 150 and N 240), more urea-^15^N moved to the deep soil for the broadcast treatment. Therefore, high rates of N fertilizer with broadcast application may increase the risk of N leaching.

### Fate of Urea-^15^N in Wheat–Soil System

**Fig 3:**
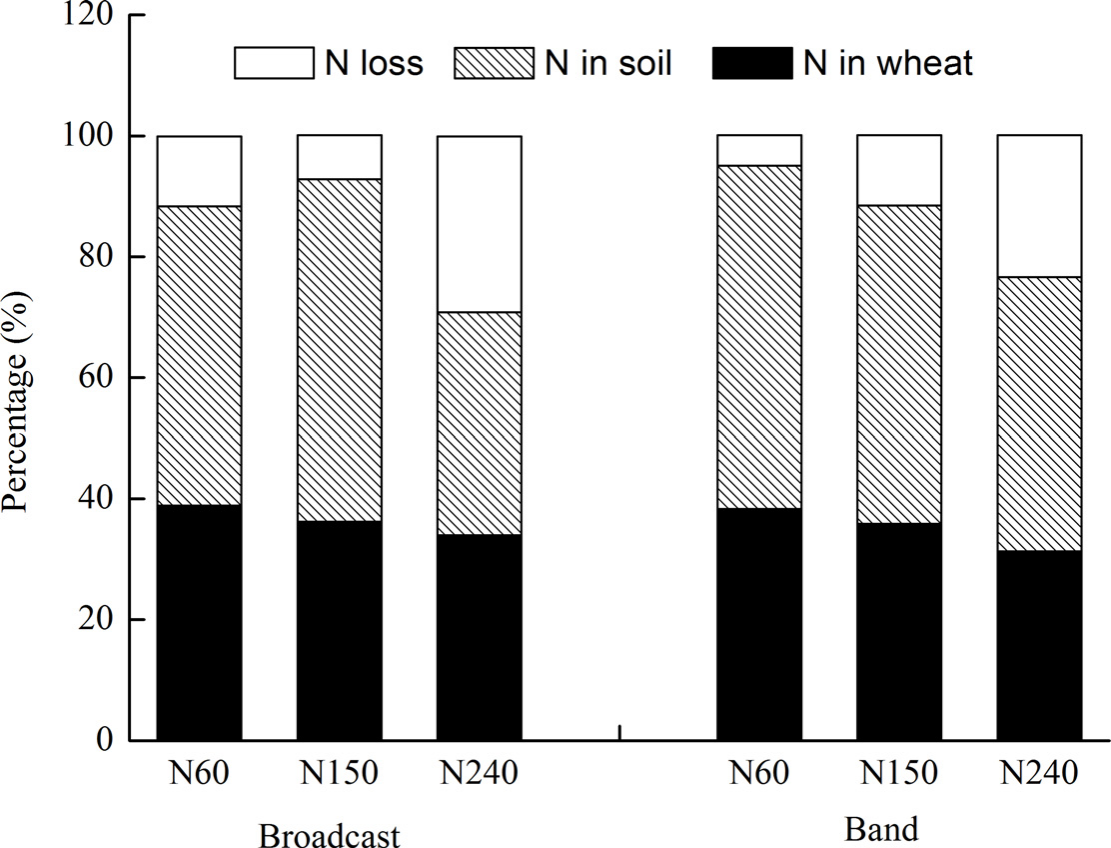
Percentages of N recoveries in wheat, and in soils (0–80 cm) and the unaccounted N losses.

The N placement method had no effect on N recovery in wheat (Table 5; Fig 3). The recovery of urea-^15^N in wheat decreased with increasing N rate. N recovery efficiency (NRE) in grain was within the range of 26.8–30.3% for broadcast and 23.6–30.6% for band treatment (Table 5). The NRE in straw was about one-fifth of that in grain. The N recovery in roots was much lower than that in grain and straw, and accounted for an average of 4% of total ^15^N recovery in wheat. The N loss increased with increasing N rate (Fig 3). For both N 60 and N 240, the unaccounted-N was significantly lower for band (5.0 and 23.5%, respectively) than for broadcast treatment (11.6 and 29.1%, respectively). The recovery in soil was within the range of 36.8–56.6% for broadcast and 45.3–56.8% for band treatment. The highest soil residual was 56.6% at N 150 for broadcast treatment, compared to 56.8% at N 60 for band treatment.

**Table 5:** Effects of N fertilizer placement and N rate on urea-^15^N recovery in wheat.

Treatment		Urea-^15^N recovery (%)			
Placement	N rate	Grain	Straw	Root	Total
Broadcast	N 60	30.3a	6.8ab	1.8a	39.0a
	N 150	29.6a	5.7b	0.9c	36.2ab
	N 240	26.8ab	6.3ab	0.9c	34.0ab
Band	N 60	30.6a	6.1ab	1.6ab	38.2a
	N 150	27.1ab	7.5a	1.3bc	35.8ab
	N 240	23.6b	6.6ab	1.1c	31.2b
Treatment effects					
Placement (P)		NS	NS	NS	NS
N rate (N)		**	NS	***	*
P×N		NS	NS	NS	NS

Different letters within a column represent significant differences (*P* < 0.05).

***, **, * Significant at *P* < 0.001, *P* < 0.01 and *P* < 0.05, respectively; NS not significant.

## Discussion

### Grain Yield Affected by Placement and N Application Rate

In our study, the grain yield of wheat was 14.3–15.4% greater for band than for broadcast treatment. Rees, et al. [32] reported that banded application of N fertilizer increased the grain yield and aboveground biomass of wheat compared with surface application in the Guanzhong Plain, Northwest China. However, grain yield was not significantly affected by placement method under field conditions in Pakistan [33]. This may be explained by the differing climate conditions, wheat cultivars and soil fertility level in these study sites. The results of the present study demonstrated that grain yield, straw yield and aboveground biomass of wheat increased with increasing N rate (*P* < 0.001), as also found by other researchers [1, 7, 26, 34]. With banded application, wheat grain yield did not differ significantly when N rate was increased from 150 to 240 kg ha^–1^. Thus, the N rate of 150 kg ha^–1^ with band application could be the recommended N management practice for winter wheat in this region—this is similar to the optimal rate of 180 kg ha^–1^ for winter wheat in southeast China [35]. The HI of wheat was lower for band than for broadcast treatment. The reason was likely partly that the higher N rate accelerated the wheat growth rate in the early stage for band treatment compared to N split-application [36].

### N Uptake Affected by Placement and N Application Rate

Our field experiment showed that increasing N application rates could improve the N concentration of winter wheat. A similar trend was also observed by Tran and Tremblay [1], who found that the N concentration increased from 22 to 28 g kg^–1^ when increasing the N application rate from 0 to 180 kg ha^–1^. The plant N concentration was higher for broadcast than for band treatment. The N concentration in grain was within the range of 15.3–22.5 g kg^–1^ for broadcast and 14.4–18.3 g kg^–1^ for band treatment. The wheat growing season is about seven months in Southeast China [26], thus the topdressing of N fertilizer would increase the grain concentration compared with a single application [37]. The placement method of N fertilizer did not affect the N uptake by grain and the whole plant, likely because band application of N fertilizer increased wheat yield but decreased the N concentration, compared with broadcast application. The whole-plant N derived from fertilizer N with broadcast and band application was within the range of 35.2–59.9% and 31.8–56.3%, respectively, while the whole-plant N derived from soil was 40.2–68.2%. This was similar to results reported by Jia et al. [26], who found that the total N uptake by wheat from soil was within the range of 45–66% in the North China Plain. Some researchers found that 15–31% of total N uptake by wheat was derived from fertilizer and the plant N uptake from soil accounted for more than 70% of total N content [38, 39]—apparently, a higher N application rate for wheat negatively affects mineralization of soil organic N. Tran and Tremblay [1] suggested that, with a low application rate of N fertilizer, soil indigenous N was important for wheat production. A micro-plot ^15^N-labeled experiment carried out in the North China Plain showed that Ndff in the total wheat plant increased with increasing N application rate, while Ndfs decreased [26].

### Fate of Urea-^15^N Affected by Placement and N Application Rate

The total N uptake by winter wheat derived from fertilizer was within the range of 18.2–64.4 kg ha^–1^ for broadcast and 18.3–56.6 kg ha^–1^ for band treatment. Rees et al. [32] found, with increasing the N rate from 75 to 150 kg ha^–1^, that the Ndfs in wheat plants increased from 20 to 37 kg ha^–1^ with surface application and from 25 to 58 kg ha^–1^ with band application. In the present study, the recovery of ^15^N in grain and wheat were within the range of 23.6–30.6% and 31.2–39.0%, respectively. Some researchers reported that the N recovery for wheat was within the range of 33.0–49.0% in the Middle and Lower Yangtze River Region [31, 40], which was higher than the results for the present study; the reason was that the wheat grain yield in our experiment was lower (5.7 vs. 6.7 t ha^–1^) due to sowing 10 d later. The N recovery is closely related to wheat cultivars, soil fertility and climate conditions [39]. With broadcast application of N fertilizer, the total N recovery in wheat was not significantly affected by the N application rate. Because the N application rate increased, and the N uptake by wheat derived from fertilizer increased correspondingly (Table 4). Similarly, Zhao et al. [40] reported that the fertilizer N recovery of wheat did not differ significantly when the N application rate increased from 100 to 250 kg ha^–1^. This trend was also observed in a micro-plot experiment in Canada in which the N application rate had no effect on the N fertilizer recovery in wheat [1]. However, the N recovery in plants was higher at low (60 kg ha^–1^) than that at high N application rate (240 kg ha^–1^) for band treatment. Ju, et al. [43] found that the N recovery in wheat was 54% at 120 kg N ha^–1^, and 32% at 360 kg N ha^–1^.

After the winter wheat was harvested, 37–57% of applied N fertilizer remained in the 0–80 cm soil layer for broadcast treatment, and 45–57% for band treatment. In a wheat–wheat rotation under Mediterranean conditions, 65% of applied-N remained in the 0–80 cm soil profile [41]. Liang et al. [3] reported that 40–80% of applied urea-N was recovered in the 0–100 cm soil layer after wheat harvest in the North China Plain. N fertilizer can move into the deep soil after wheat harvest [42]. Liang et al. [3] showed that urea-^15^N moved into the 60–100 cm soil layer and Wang et al. [34] even found that N fertilizer moved to a depth of 200 cm. In our study, the urea-^15^N in all treatments moved into the 60–80 cm soil layer (Fig 2). Nevertheless, most residual ^15^N remained in the 0–20 cm soil layer, accounting for 76–91% of total urea-N in soil. Fan et al. [4] also found that above 67% of total soil residual N was retained in the topsoil (0–20 cm) in southwest China. About 90% of the residual N can be immobilized in soil organic matter [43]. The residual N in the topsoil layer can be mineralized and then be taken up by the following crop [3, 26, 41].

Our results showed that band application reduced the unaccounted-N compared with broadcast application. The higher NH_4_^+^ concentration with band application has toxic effects on nitrifiers and then inhibits nitrification, and so increases the soil residual N [20, 21, 44]. Higher N application rate (240 kg ha^–1^) caused more N loss compared to the lower N rates (60 and 150 kg ha^–1^) irrespective of placement method. This trend was similar to the findings of Jia et al. [26] that the unaccounted-N increased from 13 to 27% when increasing the rate of N application from 150 to 270 kg ha^–1^.

The results from our study showed that N loss was lower with band application of N fertilizer; however, the N recovery in wheat was lower and the residual N was higher in the middle and lower Yangtze River basin. This indicates that the best time for band application requires further examination and banding coupled with split applications may be an alternative pattern of N management for winter wheat with a seven-month growing season.

## Conclusion

Under the field conditions of this study, band application one time of N fertilizer increased the yield of winter wheat and soil residual ^15^N, and decreased the N loss, compared to surface application three times. Nevertheless, placement method had no effect on N recovery in wheat. Split application of N fertilizer increased the grain N concentration. The yield of wheat increased with an increased N rate. At the N rate of 240 kg ha^–1^, the unaccounted-N was much higher than that at 60 and 150 kg ha^–1^. The root biomass was not affected by the placement and N rate. It was concluded that band application one time is a recommended N fertilizer management practice for winter wheat in southeast China.
